# Integrated Bioinformatics Analysis and Verification of Gene Targets for Myocardial Ischemia-Reperfusion Injury

**DOI:** 10.1155/2022/2056630

**Published:** 2022-04-15

**Authors:** Jianru Wang, Xiaohui Li, Guangcao Peng, Genhao Fan, Mengmeng Zhang, Jian Chen

**Affiliations:** ^1^Department of Cardiovascular, The First Affiliated Hospital of Henan University of CM, Zhengzhou, Henan 450099, China; ^2^Department of Vascular Disease, Shanghai TCM-Integrated Hospital, Shanghai University of Traditional Chinese Medicine, Shanghai 200082, China; ^3^Institute of Vascular Anomalies, Shanghai Academy of Traditional Chinese Medicine, Shanghai 200082, China

## Abstract

**Background:**

Myocardial ischemia-reperfusion injury (MIRI) has become a thorny and unsolved clinical problem. The pathological mechanisms of MIRI are intricate and unclear, so it is of great significance to explore potential hub genes and search for some natural products that exhibit potential therapeutic efficacy on MIRI via targeting the hub genes.

**Methods:**

First, the differential expression genes (DEGs) from GSE58486, GSE108940, and GSE115568 were screened and integrated via a robust rank aggregation algorithm. Then, the hub genes were identified and verified by the functional experiment of the MIRI mice. Finally, natural products with protective effects against MIRI were retrieved, and molecular docking simulations between hub genes and natural products were performed.

**Results:**

230 integrated DEGs and 9 hub genes were identified. After verification, Emr1, Tyrobp, Itgb2, Fcgr2b, Cybb, and Fcer1g might be the most significant genes during MIRI. A total of 75 natural products were discovered. Most of them (especially araloside C, glycyrrhizic acid, ophiopogonin D, polyphyllin I, and punicalagin) showed good ability to bind the hub genes.

**Conclusions:**

Emr1, Tyrobp, Itgb2, Fcgr2b, Cybb, and Fcer1g might be critical in the pathological process of MIRI, and the natural products (araloside C, glycyrrhizic acid, ophiopogonin D, polyphyllin I, and punicalagin) targeting these hub genes exhibited potential therapeutic efficacy on MIRI. Our findings provided new insights to explore the mechanism and treatments for MIRI and revealed new therapeutic targets for natural products with protective properties against MIRI.

## 1. Introduction

Coronary heart disease (CHD) as a global public health problem is one of the main causes of decreased quality of life and death worldwide [1]. Acute myocardial infarction (AMI) is a kind of acute and critical manifestation of CHD with high morbidity and mortality. Early rapid restoration of coronary blood flow can reduce the size of myocardial infarction and prevent further cardiac injury. With the development of reperfusion strategies such as thrombolysis and percutaneous coronary intervention, the mortality rate of AMI has been greatly reduced [2]. However, myocardial reperfusion can further cause secondary myocardial injury, which can account for 50% of the final myocardial infarct area [3]. This phenomenon, termed myocardial ischemia-reperfusion injury (MIRI), has become an unsolved clinical problem and a major cause of cardiac insufficiency. Currently, there is no specific treatment for MIRI in pharmacopeia [4]. Therefore, clinical and basic studies in the prevention and treatment of MIRI are active areas in the medical field.

It is currently believed that the pathogenesis of MIRI is a multi-factorial complex process. The latest evidence indicates that epigenetic regulation including histone modification, DNA methylation, non-coding RNAs, and N6-methyladenosine (m6A) methylation plays a key role in MIRI and can be used as new therapeutic targets for MIRI [5]. Iron, as an essential mineral, is an important player in the physiological functions of many tissues and organs in the human body. Studies in recent years have shown that imbalance in iron metabolism homeostasis is intimately related to the pathological process of MIRI [6]. Ferroptosis, a newly discovered form of regulated cell death, results from the imbalance in iron metabolism [6]. The occurrence of ferroptosis plays an important role during MIRI and can serve as a potential therapy for MIRI [7]. Furthermore, there are numerous other reported pathological mechanisms of MIRI including immunoreaction, autophagy, cell-derived exosomes, dysfunctional mitochondria, different forms of cell death, inflammation, oxidative stress, intracellular calcium overload, and so on [4, 8–10]. Although the pathological mechanisms of MIRI have been extensively explored, its specific pathogenesis has not been fully elucidated. Therefore, plentiful work is still required to better understand the potential pathologic mechanism of MIRI at the molecular level, which may be imperative to develop more effective treatments and seek early diagnostic markers.

In recent years, with the help of bioinformatics and high-throughput techniques, a comprehensive understanding of the molecular mechanism and progression of various diseases has become possible [11]. Molecular docking, a very popular and useful tool, is used to research the interaction between a small-molecule ligand and a target receptor in the drug discovery arena [12]. In our previous study, the molecular docking technique was successfully used to predict the binding modes between 12 COVID-19 targets and the 20 compounds of Qingfei Paidu decoction that have been widely used to treat COVID-19 in China [13]. The analyses of microarray and RNA-seq gene expression data by bioinformatics have been extensively used not only to explore the diagnostic and prognostic biomarkers but also to probe crucial genes and biological processes in many diseases [14–17]. However, some reasons, including the limited sample quantities, the heterogeneity of experimental samples, the use of different detection platforms, and so on, may generate deviation in the results [18, 19]. The integration of the results of multiple gene expression data sets by a robust rank aggregation (RRA) algorithm based on a statistical model that naturally allows evaluating the significance of the results is a promising strategy to overcome these shortcomings [20]. RRA has been widely used in the comprehensive analysis of multiple data sets for both oncology and non-oncology diseases [18, 19, 21]. However, there are no reports on the use of the RRA algorithm in MIRI.

**Figure 1:**
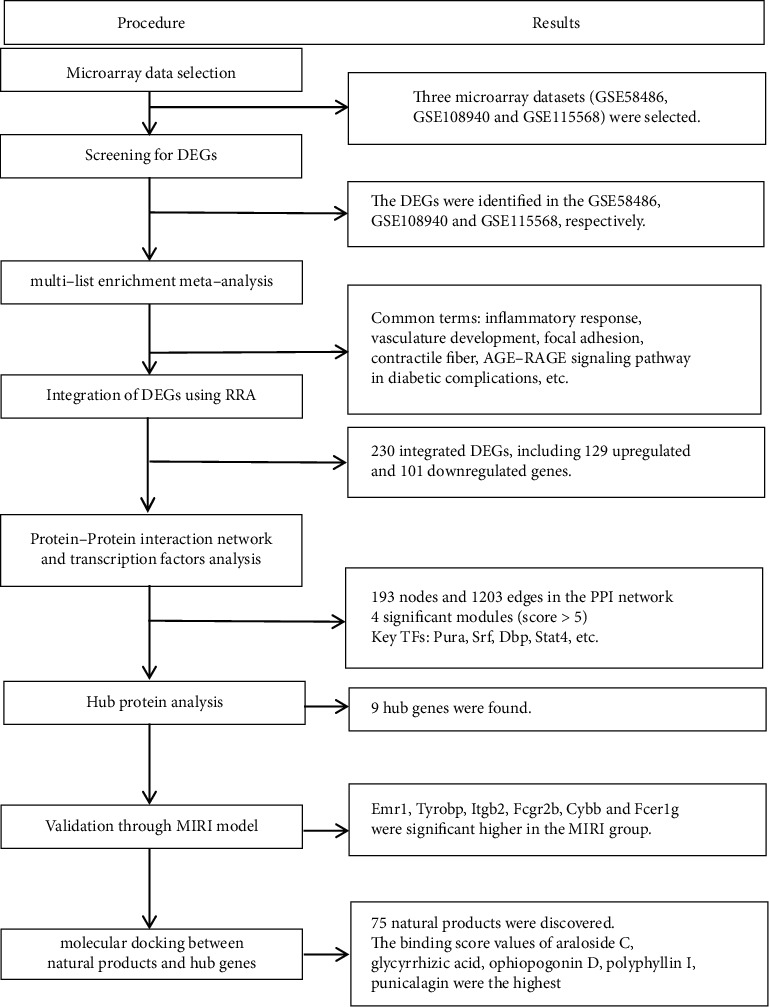
Flowchart of the study design. First, the differential expression genes (DEGs) from three arrays were screened for myocardial ischemia-reperfusion injury (MIRI) and integrated to explore the potential pathogenesis of MIRI using the RRA algorithm. Second, protein-protein interaction network and transcription factors analysis of the integrated DEGs were performed. Then, the most significant DEGs were identified and verified by the functional experiment of the MIRI mice. Finally, natural products exerting protection for MIRI were retrieved and molecular docking simulations were performed.

In this study, expression profile analyses and bioinformatics methods were combined to explore hub genes and their functions in MIRI. We subsequently performed the validation of the results via the functional experiment of the MIRI mice. Meanwhile, we retrieved the natural products in Chinese herbal medicine exerting protection for MIRI from the PubMed database. Finally, molecular docking simulations between natural products and hub genes were performed (Figure 1). To sum up, the ultimate goal of the research is to provide novel insights into new potential therapeutic targets and the pathogenesis of MIRI and develop more effective anti-MIRI drugs.

## 2. Materials and Methods

### 2.1. Affymetrix Microarray Data

We collected the expression profile data of GSE58486 (GPL18802), GSE108940 (GPL7202), and GSE115568 (GPL16570) from the Gene Expression Omnibus (GEO) database (https://www.ncbi.nlm.nih.gov/geo/). GSE58486 contains six MIRI samples and three sham surgery samples. GSE108940 contains six MIRI samples and six sham surgery samples. GSE115568 contains three MIRI samples and three sham surgery samples. All samples were collected from murine hearts.

### 2.2. Screening for Differential Expression Genes (DEGs) and Functional Enrichment Analyses

GEO2R online analysis (https://www.ncbi.nlm.nih.gov/geo/geo2r/) was firstly used to obtain the .txt files of the DEGs for the three microarray data sets. The *p*-value <0.01 and the absolute fold-change (FC) >1.5 were set to determine DEGs. Then, multi-list enrichment analysis of the three gene lists was performed using Metascape (https://metascape.org) [22]. *p* < 0.01, a minimum count of 3, and the enrichment factor >1.5 were set as the thresholds for enrichment analyses.

### 2.3. Integration of DEGs in Three Data Sets

The TXT files of all gene lists ranked by logFC in three data sets were collected for RRA analysis (https://www.icesi.edu.co/CRAN/web/packages/RobustRankAggreg/) in R version 3.5.3 (https://cran.rproject.org/), and the *p*-value <0.01 was set to define the significant integrated DEGs. The code used for the RRA analysis is presented in Supplementary Table 1. Gene ontology (GO) and pathway enrichment analyses of integrated DEGs were performed using Metascape. For pathway enrichment analyses, the KEGG pathway, Reactome gene sets, and WikiPathways were employed. The bubble plots were drawn with the ggplot2 package.

### 2.4. PPI Network and TFs Analysis

The interaction network of the integrated DEGs was performed based on the Search Tool for the Retrieval of Interacting Genes (STRING) [23] to mine their target genes, and interaction pairs with a combined score of >0.4 were considered significant. Then, Cytoscape (version 3.2.1) [24] was applied to visualize the molecular interactions. Next, MCODE analysis [25] was carried out to find densely connected modules in the PPI network. Finally, transcription factors (TFs) analysis of the MCODE modules were analyzed by the iRegulon plugin [26] of Cytoscape with the default criteria. The top five TFs of each module with the higher normalized enrichment scores (NES > 4) were listed.

### 2.5. Hub Protein Analysis

CytoHubba app [27] was used to identify the top 20 ranking genes using the degree, closeness, edge percolated component (EPC), maximum neighborhood component (MNC), and maximal clique centrality (MCC). The overlapping genes from five algorithms were taken as hub genes. At last, the functional enrichment analyses of hub genes were performed in Metascape, using GO biological processes, KEGG pathway, Reactome gene sets, and WikiPathways.

### 2.6. Establishment of MIRI Model in Mice

The animal experiments were approved by the Experimental Animal Welfare Ethics Review Committee of the First Affiliated Hospital of Henan University of CM (approval number, YFYDW2020004) and followed the National Institutes of Health Guide for the Care and Use of Laboratory Animals (NIH Publications No. 8023, revised 1978). Male C57BL/6 J mice (6–8 weeks) were purchased from Huaxing Experimental Animal Farm (Zhengzhou, China; qualified production number, SCXK-(Yu)-2019-0002). The mice were fed under a 12 h cycle of light/dark in IVC condition and had free access to food and water. Before modeling, the mice were randomly divided into two groups (*n* = 9/group), namely sham group and MIRI group. MIRI mouse model was established by ligating the left anterior descending coronary artery, as previously described [28]. After ischemia for 30 min, the ligation was released to allow reperfusion. Mice were sacrificed following reperfusion 24 h. Sham‐operated mice underwent the same procedure without ligation. Blood samples were collected for measurement of lactate dehydrogenase (LDH) activities using a kit (Nanjing Jiancheng Bioengineering Institute, Nanjing, China) following the manufacturer's instructions. The infarct area was determined by staining the heart tissues with 2% triphenyl tetrazolium chloride (TTC; Beijing Solarbio Science & Technology Co. Ltd., Beijing, China) for 15 min at 37°C. The infarct size was evaluated according to the method reported in previous literature by Image-Pro Plus 6.0 software (Media Cybernetics, Silver Spring, MD, USA) [29, 30].

### 2.7. Verifying of Hub Genes by Quantitative Real-Time Polymerase Chain Reaction (RT-qPCR)

Total RNA was extracted from each myocardial tissue using TRIzol (Invitrogen Corporation, CA, USA). cDNA was synthesized using NovoScript^®^ Plus1st Strand cDNA Synthesis SuperMix (Novoprotein Scientific Inc., Shanghai, China). RT-qPCR was performed using SYBR High-Sensitivity qPCR SuperMix (Novoprotein Scientific Inc., Shanghai, China). The primers were synthesized by Shanghai Sangon Biological Engineering Technology (Shanghai, China; Table 1). The mRNA expression level was normalized to that of *β*-actin in the same sample. The relative mRNA expression level was calculated using the 2^−ΔΔCT^ method.

### 2.8. Molecular Docking Simulation

The natural products in Chinese herbal medicine exerting protection for MIRI were retrieved from the PubMed database (https://pubmed.ncbi.nlm.nih.gov/). Then, the 2D chemical structures of natural products were obtained from PubChem (https://pubchem.ncbi.nlm.nih.gov/). The 3D structures of hub genes were obtained from the Protein Data Bank (PDB) database (https://www.rcsb.org/). Molecular docking simulation of each hub gene with natural products was performed using AutoDock Vina software.

### 2.9. Statistical Analysis

All data were presented as mean ± SEM and analyzed using IBM SPSS statistics 21.0 software. The Student's *t*-test was utilized to compare data between two groups. A *p* value < 0.05 was accepted as statistically significant.

## 3. Results

### 3.1. Identification and Functional Enrichment Analyses of DEGs

**Figure 2:**
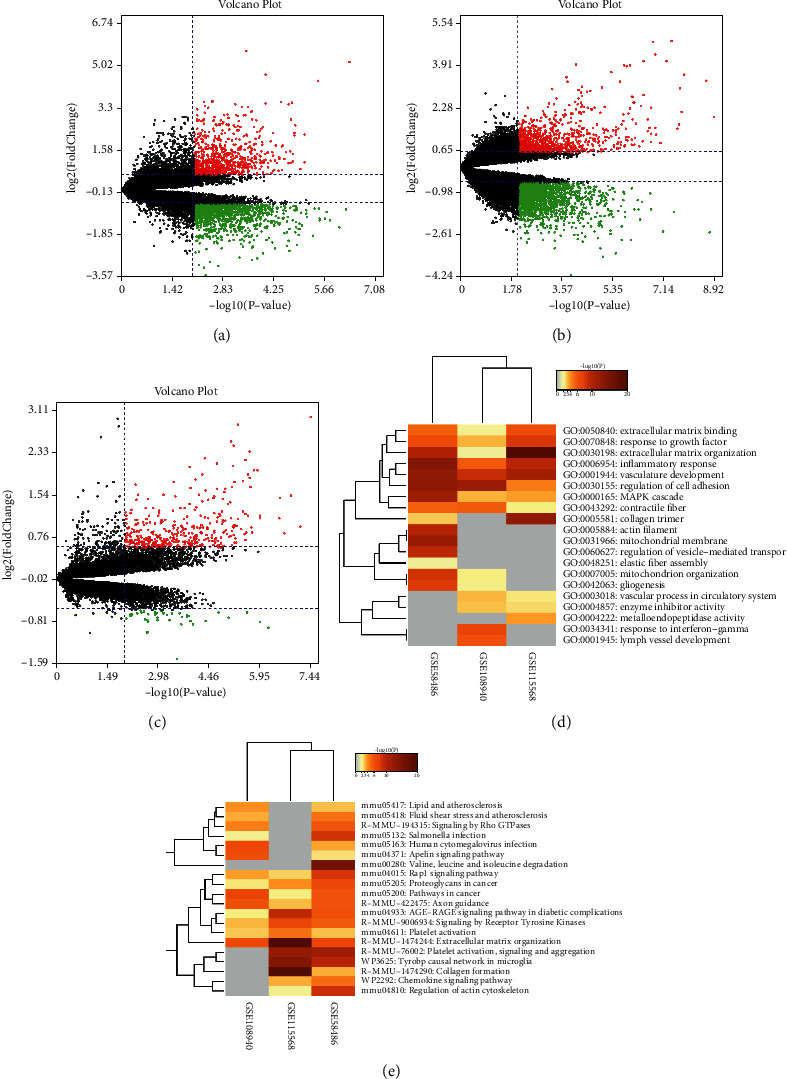
Identification and functional enrichment analyses of DEGs: (a)–(c) volcano plot for GSE58486, GSE108940, and GSE115568; (d) heatmap visualization of the results of GO enrichment analyses across multiple gene lists; and (e) heatmap visualization of the results of pathways enrichment analyses using KRGG, Reactome, and WikiPathways databases across multiple gene lists. The gray color indicates a lack of significance.

After screening with the threshold of |FC| >1.5 and *p* < 0.01, 1986, 1703, and 402 DEGs were identified in the GSE58486, GSE108940, and GSE115568, respectively (Figure 2(a)–2(c)). To facilitate the understanding of the functional mechanisms that are shared between, or selectively ascribed to, specific gene lists, a multi-gene-list meta-analysis was performed.  Figure 2(d) shows that eight GO terms were common in all studies.  Figure 2(e) shows that eight pathways were common in all studies.

### 3.2. Identification of Integrated DEGs in MIRI

**Figure 3:**
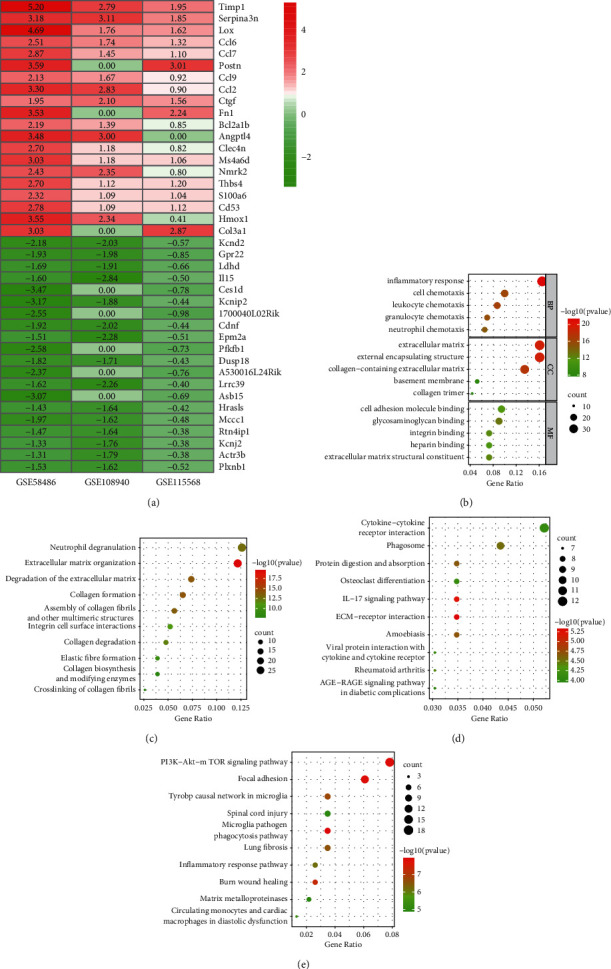
Identification and functional enrichment analyses of integrated DEGs. (a) Heatmap of top 20 up- and downregulated DEGs of three microarrays screening by RRA. Red boxes represent upregulated genes and green boxes represent downregulated genes. The values in the boxes represent logFC values. (b) The top five enriched GO terms for integrated DEGs in biological process, molecular function, and cellular component categories. (c) The top ten enriched Reactome pathways for integrated DEGs. (d): The top ten enriched KEGG pathways for integrated DEGs. (e) The top ten enriched WikiPathways for integrated DEGs.

Table 2 presents that 230 integrated DEGs were identified using the RRA (*p* < 0.01). The top 20 up- and downregulated hub genes were drawn into a heatmap (Figure 3(a)). The top five BP terms showed that integrated DEGs were mainly related to inflammatory response, cell chemotaxis, and so on (Figure 3(b)). The top five CC terms showed that integrated DEGs were mainly related to the extracellular matrix, external encapsulating structure, and so on (Figure 3(b)). The top five MF terms showed that integrated DEGs were mainly related to extracellular matrix structural constituent, cell adhesion molecule binding, and so on (Figure 3(b)). We enriched 35, 55, and 15 pathways from the KEGG, Reactome, and WikiPathways databases, respectively, setting a *p*-value < 0.01, a minimum count of 3, and an enrichment factor >1.5 as screening thresholds. The top 10 enriched Reactome pathways closely associated with MIRI were mainly neutrophil degranulation, extracellular matrix organization, collagen degradation, and so on (Figure 3(c)). The top 10 enriched KEGG pathways closely associated with MIRI were mainly IL-17 signaling pathway, AGE-RAGE signaling pathway, ECM-receptor interaction, and so on (Figure 3(d)). The top 10 enriched WikiPathways closely associated with MIRI were mainly focal adhesion, PI3K-Akt-mTOR signaling pathway, inflammatory response pathway, and so on (Figure 3(e)).

### 3.3. PPI Network and TFs Analysis

**Figure 4:**
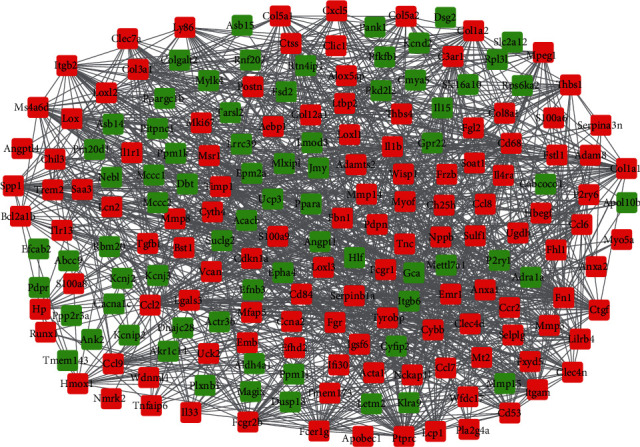
The PPI network of the 230 integrated DEGs. Red boxes represent upregulated genes, and green boxes represent downregulated genes.

**Figure 5:**
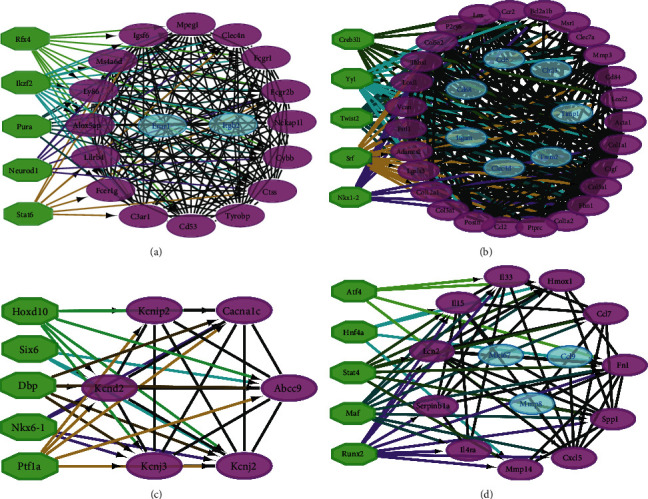
TFs analysis of MCODE modules: (a)–(d) TFs analysis of modules 1–4. Green stands for the predicted TF; purple stands for the TF-binding protein; and blue stands for the unbound protein.

There were 193 nodes and 1,203 edges in the PPI network of the 230 integrated DEGs in MIRI (Figure 4). MCODE analysis showed that four meaningful functional modules were selected (score > 5). The top 5 TFs were predicted to target these MCODE modules, including Pura, Srf, Dbp, Stat4, and so on (Figure 5(a)–5(d)).

### 3.4. Hub Protein Analysis

**Figure 6:**
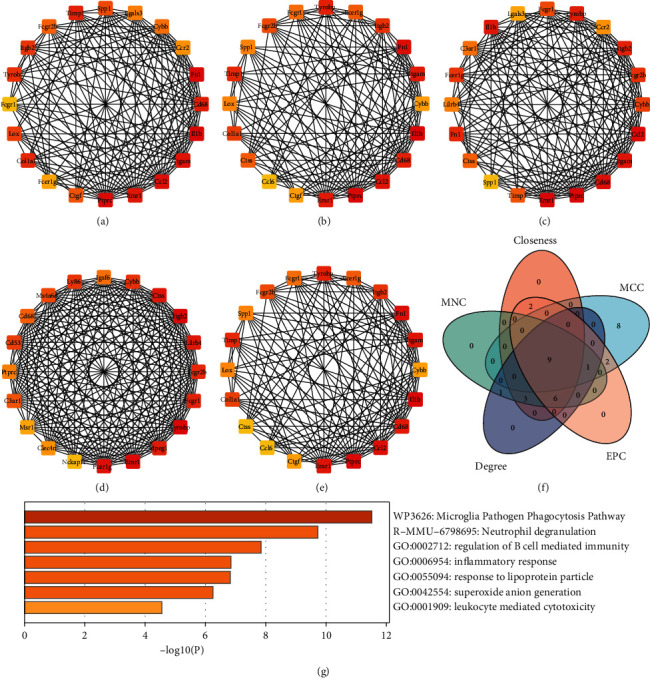
Identification and functional enrichment analysis of hub genes; (a–e) top 20 genes were calculated from the PPI network of the 230 integrated DEGs by the closeness, degree, EPC, MCC, and MNC, respectively; (f) the Venn diagram showing the hub genes identified by the CytoHubba plugin; and (g) functional enrichment analysis of the hub genes.

The hub genes were identified by CytoHubba from 230 integrated DEGs. The top 20 ranking genes, which were selected based on closeness, degree, EPC, MCC, and MNC, are displayed in Figure 6(a)–6(e). There were 9 intersection genes (Tyrobp, Itgb2, Emr1, Fcer1g, Fcgr1, Fcgr2b, Cybb, Cd68, and Ptprc) among 5 algorithms (Figure 6(f)). These genes, also known as hub genes, were selected for further validation in the MIRI model. In addition, Metascape results showed that 9 genes were mainly related to neutrophil degranulation, inflammatory response, superoxide anion generation, leukocyte mediated cytotoxicity, and so on (Figure 6(g)).

### 3.5. The Successful Establishment of the MIRI Model in Mice

**Figure 7:**
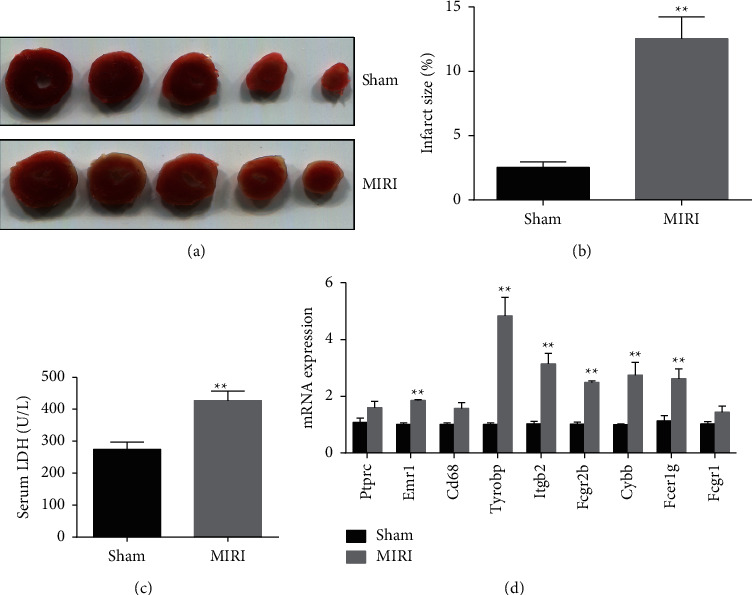
The hub genes were validated in the MIRI model: (a) representative slices stained by TTC, (b) quantitative analysis of myocardial infarct size (*n* = 5), (c) the activities of LDH in serum (*n* = 6), and (d) validation of the mRNA expression levels of hub genes (*n* = 3). ^*∗∗*^*p* < 0.01 versus the sham group.

As illustrated in Figure 7(a)–7(b), the infarct areas of hearts in the MIRI group were significantly larger than those in the sham group. Moreover, the serum LDH activities were significantly increased in mice subjected to MIRI (Figure 7(c)). Taken together, the obtained data suggested that the MIRI mouse model was successfully established.

### 3.6. The mRNA Expression Levels of Six Hub Genes Were Upregulated in the MIRI Model

To verify the results of bioinformatics analysis, we detected the mRNA expression level of hub genes by RT-qPCR in the myocardial tissues. Compared with the sham group, the mRNA expression levels of Emr1, Tyrobp, Itgb2, Fcgr2b, Cybb, and Fcer1g were higher in the MIRI group (Figure 7(d)). Unexpectedly, CD68, Ptprc, and Fcgr1 were not markedly differentially expressed. However, the changing trend was consistent with the result of bioinformatics results (Figure 7(d)).

### 3.7. Molecular Docking Results

**Figure 8:**
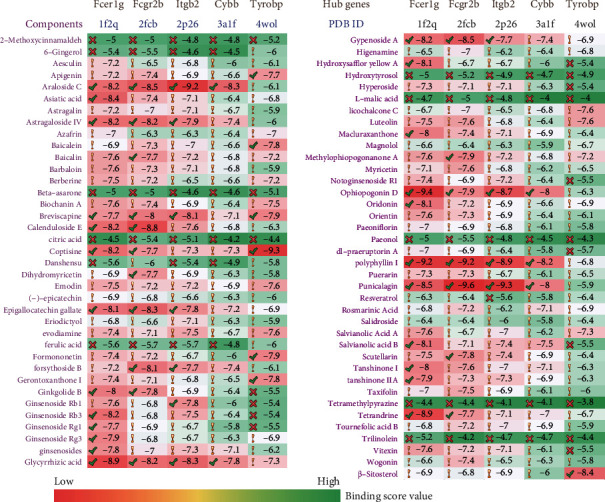
The Molecular Docking Results.

A total of 75 natural products were discovered, and their multiple mechanisms of action for treating MIRI were complex (Supplementary Table 2). To verify the effect of natural products on hub genes, molecular docking was performed. Unfortunately, the 3D structure of Emr1 was not obtained from the PDB database. The molecular docking results showed that there were different degrees of binding between natural products and hub genes, and most of the natural products showed a good ability to bind the hub genes, particularly araloside C, glycyrrhizic acid, ophiopogonin D, polyphyllin I, and punicalagin (Figure 8).

## 4. Discussion

MIRI, as an inevitable phenomenon, is a major challenge to the treatment of AMI and plays a crucial role in the damage, repair, and remodeling of myocardial tissue. MIRI not only can contribute to myocardial infarction but also can lead to myocardial stunning, the no-reflow phenomenon, reperfusion arrhythmia, lethal reperfusion injury, coronary capillary rupture, and hemorrhages [10, 31]. Despite remarkable progress in understanding the pathogenesis of MIRI, the optimal treatment strategies to effectively restrict MIRI remain elusive [32]. It has been very disappointing to translate effective strategies that have been proven in many preclinical studies into better clinical outcomes [32]. There is an undisputed unmet need between therapy and understanding the pathophysiological mechanism of MIRI. Only with a better understanding of the mechanisms of MIRI, more appropriate strategies to reduce myocardial damage can be developed. Therefore, researching the mechanisms underlying MIRI development is very important.

In this study, GSE58486, GSE108940, and GSE115568 data sets were firstly downloaded from the GEO database, and DEGs were determined. Then, the multi-gene-list meta-analyses were performed to find the common GO and KEGG terms of the DEGs. Moreover, the three data sets were analyzed using the RRA method, and integrated DEGs were found. Function annotation and pathway enrichment analyses of integrated DEGs were then conducted. The results of the above functional enrichment analyses showed that DEGs might be mainly involved in regulating the extracellular matrix, cell adhesion, cell chemotaxis, inflammatory response, AGE-RAGE signaling pathway, and PI3K-Akt-mTOR signaling pathway. MIRI induces a series of sterile inflammatory reactions, which can lead to further myocardial injury. Many risk factors, including chemokines, inflammatory cytokines, inflammatory cells, complement cascades, inflammatory pathways, and so on, play important roles in the inflammatory response during MIRI through multiple interacting pathways [33, 34]. Adhesion molecules are transmembrane glycoproteins with a variety of biological activities, which are produced by cells. They can mediate the interaction and binding between cells and cells and between cells and extracellular matrix. Adhesion molecules E-selectin and intercellular cell adhesion molecule-1 expressed on activated endothelial cells can regulate the leukocyte adhesion cascade and thus aggravate tissue inflammation in the pathological process of MIRI [35]. A study has shown that HMGB1/TLR4 signaling, as an inflammatory signaling pathway, promotes the release of inflammatory factors and aggravates MIRI by regulating the migration, adhesion, and aggregation of dendritic cells to the myocardium [36]. Substantial evidence implies the AGE-RAGE signaling pathway plays a crucial role in MIRI [37]. AGE-RAGE pathway can regulate ventricular arrhythmias, cardiomyocyte apoptosis, and contractile impairment following MIRI, which is a potential therapeutic strategy for ameliorating MIRI [37]. More recent evidence suggests that PI3K/Akt/mTOR signaling pathway protects cardiac injury from MIRI by regulating apoptosis and autophagy in cardiomyocytes [38, 39]. Thus, it can serve as a potential target in the setting of MIRI. According to the above studies, the results of the identified enrichment analyses in the current research play a recognized role in the pathogenesis of MIRI.

We also constructed a PPI network with integrated DEGs and identified the following nine hub genes: Tyrobp, Itgb2, Emr1, Fcer1g, Fcgr1, Fcgr2b, Cybb, Cd68, and Ptprc. Consistent with the bioinformatics results, MIRI mouse model results proved that Emr1, Tyrobp, Itgb2 (synonym: CD18), Fcgr2b, Cybb, and Fcer1g were significantly higher in the MIRI group. The changing trend of CD68, Ptprc, and Fcgr1 was consistent with the bioinformatics results. These results suggest that the results of our bioinformatics analysis may provide a reliable basis for the study of the mechanism of MIRI. The results of pathway and process enrichment analysis showed that hub genes were mainly related to microglia pathogen phagocytosis pathway, neutrophil degranulation, regulation of B-cell-mediated immunity, inflammatory response, response to lipoprotein particle, superoxide anion generation, leukocyte mediated cytotoxicity, and so on. Neutrophils, as the primary responders of MIRI, represent an important component in the protracted inflammatory response and severity related to MIRI [40]. Early research suggested that specific neutrophils degranulation within the ischemic myocardium occurred in MIRI [41]. In addition, superoxide anion generation that occurs during MIRI can further augment MIRI by enhancing the pro-oxidant activity of aconitase [42]. In summary, the enriched pathway and process of these hub genes were, in part, consistent with the pathological process of MIRI shown in previous studies.

It is noticeable that the role of several hub genes in MIRI has been reported in the literature. Palazzo et al. [43] found that CD18^−/−^ mice subjected to MIRI showed a marked reduction in neutrophils accumulation and myocardial necrosis. The results clearly demonstrate that deficiency of CD18 protects MIRI. Cybb expression levels were found to increase with MIRI [44]. Fortunately, myocardial ischemic postconditioning, melatonin, and RIPerC have protective effects on the myocardium by decreasing Cybb expression in the rat model of MIRI [45]. Fcer1g, adapter protein containing an immunoreceptor tyrosine-based activation motif, plays a pivotal role in the extension of MIRI at least partly through mediating collagen-induced platelet activation [46]. Tyrobp expressed in circulating immune cells is a cell membrane-associated protein, which has been reported to be involved in ischemia-reperfusion injury including the liver, lung, and kidney, but not the heart [47–49]. There are no reports on the involvement of Emr1 and Fcgr2b in MIRI. However, our findings suggest otherwise. Thus, Emr1, Tyrobp, and Fcgr2b are fascinating therapeutic targets, the inhibition of which may protect the myocardium from ischemia-reperfusion injury. Moreover, some important TFs, such as Pura, Srf, Dbp, and Stat4 were uncovered in the present study. Among these TFs, Srf has been reported to mediate MIRI by regulating its target genes (Tagln, Fos, NCX1, Slc8a1, and Egr1) [50]. To sum up, these TFs can further enhance our understanding of MIRI pathogenesis and have the potential to offer novel treatment strategies for MIRI.

Currently, there remains an unmet clinical need for the treatment methods and drugs for MIRI. Natural products especially the extracts of Chinese herbal medicine have been an important source of medicines and drug templates with proven success, which has increasingly drawn widespread attention [51, 52]. In the study, we searched the natural products in Chinese herbal medicine exerting protection for MIRI and discovered 75 natural products. To validate the therapeutic efficacy of natural products against MIRI via targeting the hub genes identified in this research, molecular docking simulations were performed. The results showed that most of the natural products showed a good binding ability to the hub genes, and the binding score values of araloside C, glycyrrhizic acid, ophiopogonin D, polyphyllin I, and punicalagin were the highest. We summarized the mechanism of action of natural products for the treatment of MIRI and found that anti-apoptosis, anti-inflammatory, anti-oxidative stress, regulating energy metabolism, reducing Ca^2+^ overload, and so on were principal mechanisms, especially araloside C, glycyrrhizic acid, ophiopogonin D, polyphyllin I, and punicalagin (Supplementary Table 2). Interestingly, the pathways and processes of the hub genes enrichment analyses were basically consistent with the mechanism of action of natural products. In summary, these natural products (especially araloside C, glycyrrhizic acid, ophiopogonin D, polyphyllin I, and punicalagin) provide a broad application prospect for developing more effective anti-MIRI drugs and will undoubtedly deserve in-depth investigation in the future.

The following are the novelties and multiple strengths of our current research. First, the RRA algorithm, a promising strategy used in the integrated analysis of multiple data sets, was first applied to explore DEGs in MIRI. Second, the functional experiment of the MIRI mice was accomplished to validate the obtained hub genes, which could avoid the bias from the results of pure bioinformatics analysis and thus yield more reliable results. Third, the molecular docking simulations of hub genes and natural products retrieved from the PubChem database were performed to analyze the potential binding effects, which could lead to better prevention and treatment for MIRI. However, our results had several limitations, which should be taken into consideration. On the one hand, our findings are based on currently available gene expression data from the microarray. Some genes, although playing potential roles in pathological processes of MIRI, were ignored in our study because they were not detected by the microarray. On the other hand, further molecular biology experiments are required to validate the function of hub genes in the progression of MIRI and the protective effects of natural products that showed a good ability to bind the hub genes against MIRI.

In conclusion, this study identified several key candidate genes, TFs, and biological pathways and searched for some natural products that exhibited potential therapeutic efficacy on MIRI via targeting the hub genes. Through our research is a preliminary investigation, the findings help us acquire a better understanding of pathogenesis, biomarkers discovery, and therapeutic targets for MIRI, reveal new therapeutic targets for natural products with protective properties against MIRI, and develop more effective anti-MIRI drugs.

## Figures and Tables

**Table 1:** Primer sequence of RT-qPCR.

Gene name	Primer sequence
*β*-actin	Forward 5'-CCCATCTACGAGGGCTAT-3'
	Reverse 5'-TGTCACGCACGATTTCC-3'
Fcer1g	Forward 5'-CGTGATCTTGTTCTTGCTCCT-3'
	Reverse 5'-TTCGGACCTGGATCTTGAGT-3'
Cybb	Forward 5'-GCTATGAGGTGGTGATGTTAGT-3'
	Reverse 5'-GCTGAGGAAGTTGGCATTGT-3'
Fcgr2b	Forward 5'-AATCCTGCCGTTCCTACTGAT-3'
	Reverse 5'-AGTGTCACCGTGTCTTCCTT-3'
Cd68	Forward 5'-CCTTCACGATGACACCTACAG-3'
	Reverse 5'-AACAGTGGAGGATCTTGGACTA-3'
Itgb2	Forward 5'-TGTCCTCCTCCTGGTCATCT-3'
	Reverse 5'-CCGTTGTCGTAGCACTCTTG-3'
Ptprc	Forward 5'-GGTTGTTCTGTGCCTTGTTCA-3'
	Reverse 5'-ATCCTGCTTGCCTCCATCC-3'
Emr1	Forward 5'-CAGGAGTGGAATGTCAAGATGT-3'
	Reverse 5'-CACAGAGTTAGAGCAGTTGGAA-3'
Tyrobp	Forward 5'-TCTGTTCCTTCCTGTCCTCCT-3'
	Reverse 5'-CTCAGTCTCAGCAATGTGTTGT-3'
Fcgr1	Forward 5'-TCAGGACAGTGGCGAATACAG-3'
	Reverse 5'-GAATGGCGACCTCCGAATCT-3'

**Table 2:** Integrated DEGs in MIRI by RRA.

DEGs	Gene names
Upregulated	Timp1, Serpina3n, Lox, Ccl6, Ccl7, Postn, Ccl9, Ccl2, Ctgf, Fn1, Bcl2a1b, Angptl4, Clec4n, Ms4a6d, Nmrk2, Thbs4, S100a6, Cd53, Hmox1, Col3a1, Adam8, Emp1, Ctss, Ltbp2, Sprr1a, Spp1, Cdkn1a, Col1a1, Mmp14, Aldh1a2, Aebp1, Lcn2, Ugdh, Fxyd5, Efhd2, Mpeg1, Lgals3, Col8a1, Lilrb4, Il4ra, Ccl8, Il33, Uck2, Fgl2, Ptprc, Ccr2, Tmem173, Car13, Mt2, Tnc, Snora75, Hp, Fcgr2b, Ch25h, Myo5a, Col1a2, Anxa1, Fcer1g, Fbn1, Alox5ap, Col5a2, Clic1, Thbs1, Ly86, Il1r1, Saa3, Cd68, Cybb, Pla2g4a, Tyrobp, Clec4d, Cxcl5, Nckap1l, Myof, Ifi30, Igsf6, Pdpn, Cd84, Vcan, Emr1, Chil3, Lcp1, Loxl1, P2ry6, Fstl1, Tnfaip6, Col12a1, Tlr13, Adamts2, Fcgr1, Mmp3, Il1b, Selplg, Col5a1, Clec7a, Mki67, Cyth4, Sulf1, C3ar1, Wisp1, Anxa2, Mfap5, Frzb, Apobec1, Nppb, Itgb2, Serpinb1a, Trem2, Ccna2, Loxl2, Retnlg, Loxl3, Soat1, Itgam, Eif1a, Tgfbi, S100a8, Hbegf, Wfdc17, Runx1, S100a9, Fgr, Emb, Bst1, Mmp8, Msr1, Fhl1, Acta1, Wfdc21
Downregulated	Kcnd2, Gpr22, Ldhd, Il15, Ces1d, Kcnip2, 1700040L02Rik, Cdnf, Epm2a, Pfkfb1, Dusp18, A530016L24Rik, Lrrc39, Asb15, Hrasls, Mccc1, Rtn4ip1, Kcnj2, Actr3b, Plxnb1, Tarsl2, Letm2, Fsd2, P2ry1, Dnajc28, Intu, Kcnv2, Ccdc141, Magix, Asb14, Rbm20, Kcnj3, Cmya5, Acacb, Angpt1, Suclg2, Dbt, Wnk2, Rpl3l, Abcc9, Gm10336, Efcab2, Cpeb3, A930018M24Rik, Gca, Slc38a3, Adra1a, Itgb6, Ppm1k, Pdpr, Mylk4, Mettl7a1, Arhgap20, Rnf207, Krt222, Mccc2, Gm31251, Klf9, Epha4, Tcp11l2, Aldh4a1, Efnb3, Akr1c14, Ppp2r3a, Tmem143, Isoc2a, Btnl9, Fyco1, Jmy, Helt, Tcaim, Pank1, Osbpl3, Slc16a10, Klra21, Pkd2l2, Cacna1c, Osbp2, Ppm1l, Cyfip2, Hlf, Gm30307, Lmod3, Slc2a12, Nebl, Pm20d1, Ppara, Mmp15, C1qtnf9, Tmem25, Ank2, Mitf, Colgalt2, Pitpnc1, Mlxipl, Ppargc1b, Lrtm1, Rps6ka2, Apol10b, Dsg2, Ucp3

## Data Availability

GSE58486, GSE108940, and GSE115568 data sets used in this study were downloaded from the GEO database (https://www.ncbi.nlm.nih.gov/geo). The data used to support the findings of this study are included in the article.

## Supplementary Materials

Supplementary Materials Supplementary Table 1. The R code used for the RRA analyses. Supplementary Table 2. Summary of natural products in Chinese herbal medicine exerting protection for MIRI.
