# Supplementary material for: Integrated Bioinformatics Analysis and Verification of Gene Targets for Myocardial Ischemia-Reperfusion Injury
Source: Evid Based Complement Alternat Med. 2022 Apr 15;2022:2056630. doi: 10.1155/2022/2056630 (PMC9033367; doi:10.1155/2022/2056630)
Supplement: Supplementary Materials — Supplementary Table 1. The R code used for the RRA analyses. Supplementary Table 2. Summary of natural products in Chinese herbal medicine exerting protection for MIRI. [file 2056630.f1.zip › 2056630.f1/Supplementary Table 1 (1).doc]

######install.packages("RobustRankAggreg")

setwd("C:\\Users\\Administrator\\Desktop\\MIRI\\RobustRankAggreg_analysis")

padj=0.01 #其实是P值

logFC=1

files=c("GSE58486.txt","GSE108940.txt","GSE115568.txt")

upList=list()

downList=list()

allFCList=list()

for(i in 1:length(files)){

inputFile=files[i]

rt=read.table(inputFile,header=T)

header=unlist(strsplit(inputFile,"\\."))

downList[[header[1]]]=as.vector(rt[,1])

upList[[header[1]]]=rev(as.vector(rt[,1]))

fcCol=rt[,1:2]

colnames(fcCol)=c("Gene",header[[1]])

allFCList[[header[1]]]=fcCol

}

mergeLe=function(x,y){

merge(x,y,by="Gene",all=T)}

newTab=Reduce(mergeLe,allFCList)

rownames(newTab)=newTab[,1]

newTab=newTab[,2:ncol(newTab)]

newTab[is.na(newTab)]=0

library(RobustRankAggreg)

upMatrix = rankMatrix(upList)

upAR = aggregateRanks(rmat=upMatrix)

colnames(upAR)=c("Name","Pvalue")

upAdj=p.adjust(upAR$Pvalue,method="bonferroni")

upXls=cbind(upAR,adjPvalue=upAdj)

upFC=newTab[as.vector(upXls[,1]),]

upXls=cbind(upXls,logFC=rowMeans(upFC))

write.table(upXls,file="up.xls",sep="\t",quote=F,row.names=F)

upSig=upXls[(upXls$Pvalue<padj & upXls$logFC>logFC),]

write.table(upSig,file="upSig.xls",sep="\t",quote=F,row.names=F)

downMatrix = rankMatrix(downList)

downAR = aggregateRanks(rmat=downMatrix)

colnames(downAR)=c("Name","Pvalue")

downAdj=p.adjust(downAR$Pvalue,method="bonferroni")

downXls=cbind(downAR,adjPvalue=downAdj)

downFC=newTab[as.vector(downXls[,1]),]

downXls=cbind(downXls,logFC=rowMeans(downFC))

write.table(downXls,file="down.xls",sep="\t",quote=F,row.names=F)

downSig=downXls[(downXls$Pvalue<padj & downXls$logFC< -logFC),]

write.table(downSig,file="downSig.xls",sep="\t",quote=F,row.names=F)

#logFC.tiff

hminput=newTab[c(as.vector(upSig[1:20,1]),as.vector(downSig[1:20,1])),]

library(pheatmap)

tiff(file="logFC.tiff",width = 15,height = 20,units ="cm",compression="lzw",bg="white",res=400)

pheatmap(hminput,display_numbers = TRUE,fontsize_row=10,fontsize_col=12,

color = colorRampPalette(c("green", "white", "red"))(50),

cluster_cols = FALSE,cluster_rows = FALSE, )

dev.off()
