# Supplementary material for: Integrated Bioinformatics Analysis and Verification of Gene Targets for Myocardial Ischemia-Reperfusion Injury
Source: Evid Based Complement Alternat Med. 2022 Apr 15;2022:2056630. doi: 10.1155/2022/2056630 (PMC9033367; doi:10.1155/2022/2056630)
Supplement: Supplementary Materials — Supplementary Table 1. The R code used for the RRA analyses. Supplementary Table 2. Summary of natural products in Chinese herbal medicine exerting protection for MIRI. [file 2056630.f1.zip › 2056630.f1/Supplementary Table 2.doc]

| Supplementary Table 2 summary of natural products in Chinese herbal medicine exerting protection for MIRI. | | | |
| --- | --- | --- | --- |
| **Natural products** | **source** | **mechanism** | **Model and Reference** |
| Trilinolein | panax pseudoginseng | Antioxidant | Isolated Rat HR model (ex vivo)[1, 2] |
| Notoginsenoside R1 | Panax notoginseng | Upregulation of miR-132; inhibiting ROCK and enhancing mitochondrial ATP synthase δ-subunits; suppressing oxidative stress- and ERS- associated apoptosis. | H9c2 cardiomyocytes HR model (in vitro)[3];  Rat MIRI model (in vivo)[4];  H9c2 cardiomyocytes HR model (in vitro) and Isolated Rat HR model (ex vivo)[5] |
| β-Sitosterol | Widely distributed in plants | Modulating the PPAR*γ*/NF-*κ*B signalling | H9c2 cardiomyocytes HR model (in vitro) and Rat MIRI model (in vivo)[6] |
| (−)-epicatechin | [Ginkgo Semen](https://old.tcmsp-e.com/tcmspsearch.php?qr=Ginkgo Semen&qsr="herb_en_name"&token=d7ed092ebbdf4d60925c84036c1f65ef), [Drynariae Rhizoma](https://old.tcmsp-e.com/tcmspsearch.php?qr=Drynariae Rhizoma&qsr="herb_en_name"&token=d7ed092ebbdf4d60925c84036c1f65ef), etc. | Preservation of mitochondrial function through enhanced substrate provision | Rat MIRI model (in vivo)[7] |
| Wogonin | Scutellaria baicalensis Georgi | Antioxidant and anti-inflammatory | Rat MIRI model (in vivo)[8] |
| Vitexin | Anthurium versicolor, Cucumis sativus L. (Cucurbitaceae), and Acer palmatum (Aceraceae) | Regulating mitochondrial dysfunction;  regulating inflammatory cytokines and MAPK pathway; inhibiting myocardial apoptosis and lipid peroxidation. | H9c2 cardiomyocytes HR model (in vitro) and Rat MIRI model (in vivo)[9-12] |
| Tournefolic acid B | Clinopodium chinense (Benth.) Kuntze | Inhibiting endoplasmic reticulum stress-regulated apoptosis via PI3K/AKT pathways | H9c2 cardiomyocytes HR model (in vitro) and Rat MIRI model (in vivo)[13] |
| Tetrandrine | root of Stephania tetrandra S Moore | Enhancing glycolysis by targeting JAK3/STAT3/HKII | H9c2 cardiomyocytes HR model (in vitro)[14] |
| Tetramethylpyrazine | [Chuanxiong Rhizoma](https://old.tcmsp-e.com/tcmspsearch.php?qr=Chuanxiong Rhizoma&qsr="herb_en_name"&token=d7ed092ebbdf4d60925c84036c1f65ef) | Antioxidant; maintain cellular Ca^2+^ homeostasis; anti-inflammatory; anti-apoptosis | Rat MIRI model (in vivo)[15] |
| Taxifolin | [Smilax China](https://old.tcmsp-e.com/tcmspsearch.php?qr=Smilax China&qsr="herb_en_name"&token=d7ed092ebbdf4d60925c84036c1f65ef), [Cinnamomi Ramulus](https://old.tcmsp-e.com/tcmspsearch.php?qr=Cinnamomi Ramulus&qsr="herb_en_name"&token=d7ed092ebbdf4d60925c84036c1f65ef), etc. | Modulating mitochondrial apoptosis pathway | Rat MIRI model (in vivo)[16] |
| tanshinone IIA | Salvia miltiorrhiza Bunge | Decreasing cardiac apoptosis and inflammation by the PI3K/Akt-dependent pathway. | Diabetic rat MIRI model (in vivo)[17] |
| Tanshinone I | Salvia miltiorrhiza Bunge | Antioxidant and alleviating necroptosis. | H9c2 cardiomyocytes HR model (in vitro) and Rat MIRI model (in vivo)[19] |
| Scutellarin | Erigeron breviscapus | Reducing apoptosis, oxidative stress and inflammatory response. | H9c2 cardiomyocytes HR model (in vitro) and Rat MIRI model (in vivo)[20, 21] |
| Salvianolic acid B | Salvia miltiorrhiza Bunge | Decreased inflammatory responses and apoptosis . | Rat MIRI model (in vivo)[22] |
| Salvianolic Acid A | Salvia miltiorrhiza Bunge | Reducing apoptosis and oxidative stress | H9c2 cardiomyocytes HR model (in vitro) and Rat MIRI model (in vivo)[23]; Isolated Rat HR model (ex vivo)[24] |
| Salidroside | Rhodiola rosea L. | Alleviating endoplasmic reticulum stress or endoplasmic reticulum stress-induced apoptosis | H9c2 cardiomyocytes HR model (in vitro)[25] |
| Rosmarinic Acid | Labiatae herbs | Inhibiting inflammation and cardiomyocyte apoptosis. | Rat MIRI model (in vivo)[26] |
| Resveratrol | found in grapes, berries, and other plants | Antioxidative stress, anti-inflammatory, anti-apoptosis and inhibiting intracellular Ca^2+^ accumulation | Rat MIRI model (in vivo)[27, 28]; H9c2 cardiomyocytes AR model (in vitro)[29]; H9c2 cardiomyocytes HR model (in vitro)[28, 30, 31]; Isolated Rat HR model (ex vivo)[31] |
| Punicalagin | pomegranate juice | Antioxidative stress | Rat MIRI model (in vivo)[32] |
| Puerarin | kudzu root | Inhibiting apoptosis and oxidative stress | H9c2 cardiomyocytes HR model (in vitro)[33] |
| polyphyllin I | rhizome of Paris polyphylla | Inhibiting the inflammatory response and oxidative stress. | Rat MIRI model (in vivo)[34] |
| Paeonol | the root bark of Paeonia × suffruticosa Andrews；the root of Paeonia lactiflora Pall；Moutan Cortex | Inhibiting apoptosis and autophagic cell death via mediating apoptosis and autophagy crosstalk. | Rat MIRI model (in vivo)[36] |
| Paeoniflorin | Paeonia albiflora | Reducing oxidative stress and apoptosis | Rat MIRI model (in vivo)[37] |
| Orientin | Polygonum orientale L. | Maintaining balance of autophagy | Isolated Rat HR model (ex vivo)[38] |
| Oridonin | rabdosia rubescens | Inhibiting the oxidative stress and NLRP3 inflammasome pathway | Mice MIRI model (in vivo)[39] |
| Ophiopogonin D | Radix Ophiopogon japonicus | Antioxidant and anti- apoptosis | Rat MIRI model (in vivo)[40] |
| Emodin | the rhizome of Rheum palmatum | Inhibiting gasdermin D-mediated pyroptosis. | Rat primary cardiomyocytes HR model (ex vivo) and Rat MIRI model (in vivo)[41] |
| Myricetin | found abundantly in numerous plants such as tea leaves, berries, fruits, vegetables, and medicinal herbs. | Anti-inflammatory, antioxidant and anti- apoptosis | Isolated Rat HR model (ex vivo)[42] |
| Methylophiopogonanone A | The dried tuber root of Ophiopogon japonicus | Anti-apoptosis | Mice MIRI model (in vivo)[43] |
| Magnolol | Magnolia officinalis | Anti-apoptosis | Rat MIRI model (in vivo)[44] |
| Luteolin | found in many fruits, vegetables and medicinal herbs | Anti-inflammatory; anti-apoptosis | Rat MIRI model (in vivo) and H9c2 cardiomyocytes HR model (in vitro)[45, 46] |
| licochalcone C | Licorice | anti-inflammatory; anti-apoptosis; antioxidant | Isolated Rat HR model (ex vivo)[47] |
| Hyperoside | Hypericum attenuatum Chois | anti-apoptosis | Rat primary cardiomyocytes HR model (ex vivo)[48] |
| Hydroxytyrosol | olive oil | anti-apoptosis; antioxidant | Rat MIRI model (in vivo)[49] |
| Hydroxysafflor yellow A | Carthami flos (Carthamus tinctorius L.) | Suppressing NLRP3 inflammasome; activating autophagy; inhibiting calcium overload and myocardial apoptosis. | Rat MIRI model (in vivo)[50]; H9c2 cardiomyocytes HR model (in vitro)[51]; Rat primary cardiomyocytes HR model (ex vivo) and Rat MIRI model (in vivo)[52] |
| Higenamine | aconite root | Anti-apoptosis | Isolated Mice HR model (ex vivo) and Mice MIRI model (in vivo)[53] |
| Gypenoside A | Gynostemma pentaphyllum | Anti-apoptosis | H9c2 cardiomyocytes OGD/R model (in vitro) and Rat MIRI model (in vivo)[54] |
| ferulic acid | Anglica sinensis (olivi) Didl. and Ligusticum chuoanxiong Hort. | Improving myocardial viability, reducing the amount of LDH leak, increasing the activity of Na+‑K+‑ATPase and Ca2+‑ATPase. | Rat primary cardiomyocytes HR model (ex vivo)[55] |
| Glycyrrhizic acid | licorice plants (Glycyrrhiza glabra) | Inhibiting endoplasmic reticulum stress | Rat MIRI model (in vivo) and H9c2 cardiomyocytes HR model (in vitro)[56] |
| ginsenosides | Panax ginseng | Increasing the release of prostacyclin, and decreasing lipid peroxidation | Dog MIRI model (in vivo)[57] |
| Ginsenoside Rg3 | ginseng | Antioxidative, anti-inflammation and [anti-fibrosis](javascript:;) | Rat MIRI model (in vivo) and H9c2 cardiomyocytes HR model (in vitro)[58] |
| Ginsenoside Rb3 | Panax ginseng | Antioxidant and improving microcirculatory disturbance. | Rat MIRI model (in vivo)[59] |
| Ginsenoside Rg1 | Radix ginseng | Inhibiting myocardial apoptosis and modulating energy metabolism | Rat MIRI model (in vivo)[60] |
| Ginsenoside Rb1 | Panax ginseng C. A. Mey | Inhibiting myocardial apoptosis and modulating energy metabolism | Rat MIRI model (in vivo)[61] |
| Gerontoxanthone I | Garcinia bracteata C. Y. Wu ex Y. H. Li. | Inducing PINK1-Parkin–mediated mitophagy; decreasing cell death and the level of ROS. | H9c2 cardiomyocytes HR model (in vitro)[62] |
| Macluraxanthone | Garcinia bracteata C. Y. Wu ex Y. H. Li. | Inducing PINK1-Parkin–mediated mitophagy; decreasing cell death and the level of ROS. | H9c2 cardiomyocytes HR model (in vitro)[62] |
| forsythoside B | the leaves of Lamiophlomis rotata Kudo. | Antioxidative, anti-inflammation | Rat MIRI model (in vivo)[63] |
| Formononetin | Radix Astragali | Enhancing autophagy | H9c2 cardiomyocytes HR model (in vitro) and Isolated mice HR model (ex vivo)[64] |
| Ginkgolide B | Ginkgo biloba | Anti-apoptosis | Isolated rat HR model (ex vivo)[65] |
| evodiamine | evodia rutaecarpa bentham (rutaceae) | Stimulating endogenous calcitonin gene-related peptide release by activation of vanilloid receptors. | Rat MIRI model (in vivo)[66] |
| Eriodictyol | present in fruits and vegetables, especially citrus such as lemon | Anti-inflammation and anti-apoptosis | Rat MIRI model (in vivo)[67] |
| Epigallocatechin gallate | [Ginkgo Semen](https://old.tcmsp-e.com/tcmspsearch.php?qr=Ginkgo Semen&qsr="herb_en_name"&token=bc8354cc881c02efb7cc22db6636c085), [Eriobotryae Folium](https://old.tcmsp-e.com/tcmspsearch.php?qr=Eriobotryae Folium&qsr="herb_en_name"&token=bc8354cc881c02efb7cc22db6636c085), [Phyllanthi Fructus](https://old.tcmsp-e.com/tcmspsearch.php?qr=Phyllanthi Fructus&qsr="herb_en_name"&token=bc8354cc881c02efb7cc22db6636c085) | Reducing cytochrome c release and cellular apoptosis | Isolated Mice HR model (ex vivo) and Mice MIRI model (in vivo)[68] |
| dl-praeruptorin A (Pd-Ia) | [Peucedani Radix](https://old.tcmsp-e.com/tcmspsearch.php?qr=Peucedani Radix&qsr="herb_en_name"&token=bc8354cc881c02efb7cc22db6636c085) | Anti-inflammation and anti-apoptosis | Rat MIRI model (in vivo)[69] |
| Asiatic acid | Centella Asiatica | Protecting cardiomyocytes from ROS‑mediated autophagy | H9c2 cardiomyocytes HR model (in vitro) and mice MIRI model (in vivo)[70] |
| Dihydromyricetin | Ampelopsis grossedentata | Improving mitochondrial function and decreased oxidative stress. | Mice primary cardiomyocytes HR model (ex vivo) and mice MIRI model (in vivo)[71] |
| Danshensu | Salvia miltiorrhiza (Danshen) | Inhibiting autophagy and apoptosis, antioxidant | Isolated rat HR model (ex vivo)[72, 73] |
| Coptisine | Coptidis Rhizoma | Suppressing autophagy, anti apoptosis | H9c2 cardiomyocytes HR model (in vitro) and rat MIRI model (in vivo)[74, 75] |
| Astragaloside IV | Astragalus membranaceus | Antioxidative, anti-apoptosis, regulating energy metabolism, suppressing autophagy | Isolated rat HR model (ex vivo)[76, 77], rat MIRI model (in vivo)[76, 78], rat primary cardiomyocytes HR model (ex vivo)[77, 79], AC16 cell HR model (in vitro)[80], H9c2 cardiomyocytes HR model (in vitro)[81]. |
| citric acid | Fructus Choerospondiatis | Anti-inflammatory, anti-platelet aggregation, anti-apoptosis | rat primary cardiomyocytes HR model (ex vivo) and rat MIRI model (in vivo)[82] |
| L-malic acid | Fructus Choerospondiatis | Anti-inflammatory, anti-platelet aggregation, anti-apoptosis | rat primary cardiomyocytes HR model (ex vivo) and rat MIRI model (in vivo)[82] |
| Calenduloside E | Aralia elata (Miq.) | Restoring mitochondrial homeostasis | H9c2 cardiomyocytes HR model (in vitro) and rat MIRI model (in vivo)[83] |
| Breviscapine | Erigeron breviscapus | Increasing ATPase activities, decreasing decreased oxidative stress and the intercellular adhesion molecule-1(ICAM-1) protein expression. | Diabetic rat MIRI model (in vivo)[84] |
| Biochanin A | Trifolium pratense L. | Anti-inflammatory | rat MIRI model (in vivo)[85] |
| Beta-asarone | Acorus tatarinowii Rhizoma | Inhibiting Inflammatory and Pyroptosis | rat MIRI model (in vivo)[86] |
| Berberine | Coptis chinensis (Huanglian) | Anti-inflammatory | rat MIRI model (in vivo)[87] |
| Barbaloin | aloe vera | Anti-inflammatory, antioxidant | rat MIRI model (in vivo)[88] |
| Baicalin | Scutellaria baicalensis Georgi | Anti-apoptosis | H9c2 cardiomyocytes HR model (in vitro)[89] |
| Baicalein | Scutellaria baicalensis Georgi | Anti-inflammatory, antioxidant, anti-apoptosis | mice MIRI model (in vivo) and rat primary cardiomyocytes HR model (ex vivo)[90] |
| Azafrin | Centranthera grandiflora | Antioxidant | H9c2 cardiomyocytes HR model (in vitro) and rat MIRI model (in vivo)[91] |
| Astragalin | leaves of persimmon, Rosa agrestis, or green tea seeds | Anti-inflammatory, antioxidant, anti-apoptosis | Isolated rat HR model (ex vivo)[92] |
| Araloside C | Aralia elata (Miq) Seem | Antioxidant, reducing Ca2+ overload | Isolated rat HR model (ex vivo) and H9c2 cardiomyocytes HR model (in vitro)[93] |
| Apigenin | present in fruits, vegetables, parsley, celery, celeriac, and chamomile tea | Anti-inflammatory, anti-apoptosis, reducing pyroptosis | H9c2 cardiomyocytes HR model (in vitro)[94-96], rat MIRI model (in vivo)[94] |
| Aesculin | Fraxinus rhynchophylla Hance | Suppressing pyroptosis | rat primary cardiomyocytes HR model (ex vivo) and rat MIRI model (in vivo) [97] |
| 6-Gingerol | ginger | Anti-inflammatory, anti-apoptosis | rat MIRI model (in vivo)[98, 99], AC16 cell HR model (in vitro)[100] |
| 2-Methoxycinnamaldehyde | Cinnamomum cassia | Antioxidant, anti-inflammatory | rat MIRI model (in vivo)[101] |
| Myocardial ischemia-reperfusion injury (MIRI); Hypoxia reoxygenation (HR); oxygen–glucose deprivation/re-oxygenation (OGD/R) | | | |

**Reference**

[1.]	P. Chan and B. Tomlinson. "Antioxidant effects of Chinese traditional medicine: focus on trilinolein isolated from the Chinese herb sanchi (Panax pseudoginseng)," *J Clin Pharmacol, vol.* 40, no. 5, pp. 457-461.

[2.]	P. Chan, C.S. Niu, B. Tomlinson, et al. "Effect of trilinolein on superoxide dismutase activity and left ventricular pressure in isolated rat hearts subjected to hypoxia and normoxic perfusion," *Pharmacology, vol.* 55, no. 5, pp. 252-258.

[3.]	Z. Jin, C. Gan, G. Luo, et al. "Notoginsenoside R1 protects hypoxia-reoxygenation deprivation-induced injury by upregulation of miR-132 in H9c2 cells," *Hum Exp Toxicol*, p. 9603271211025589.

[4.]	K. He, L. Yan, C.S. Pan, et al. "ROCK-dependent ATP5D modulation contributes to the protection of notoginsenoside NR1 against ischemia-reperfusion-induced myocardial injury," *Am J Physiol Heart Circ Physiol, vol.* 307, no. 12, pp. H1764-1776.

[5.]	Y. Yu, G. Sun, Y. Luo, et al. "Cardioprotective effects of Notoginsenoside R1 against ischemia/reperfusion injuries by regulating oxidative stress- and endoplasmic reticulum stress- related signaling pathways," *Sci Rep, vol.* 6, p. 21730.

[6.]	F. Lin, L. Xu, M. Huang, et al. "β-Sitosterol Protects against Myocardial Ischemia/Reperfusion Injury via Targeting PPARγ/NF-κB Signalling," *Evid Based Complement Alternat Med, vol.* 2020, p. 2679409.

[7.]	K.G. Yamazaki, A.Y. Andreyev, P. Ortiz-Vilchis, et al. "Intravenous (-)-epicatechin reduces myocardial ischemic injury by protecting mitochondrial function," *Int J Cardiol, vol.* 175, no. 2, pp. 297-306.

[8.]	Y.M. Lee, P.Y. Cheng, S.Y. Chen, M.T. Chung, and J.R. Sheu. "Wogonin suppresses arrhythmias, inflammatory responses, and apoptosis induced by myocardial ischemia/reperfusion in rats," *J Cardiovasc Pharmacol, vol.* 58, no. 2, pp. 133-142.

[9.]	H. Yang, W. Xue, C. Ding, et al. "Vitexin Mitigates Myocardial Ischemia/Reperfusion Injury in Rats by Regulating Mitochondrial Dysfunction via Epac1-Rap1 Signaling," *Oxid Med Cell Longev, vol.* 2021, p. 9921982.

[10.]	W. Xue, X. Wang, H. Tang, et al. "Vitexin attenuates myocardial ischemia/reperfusion injury in rats by regulating mitochondrial dysfunction induced by mitochondrial dynamics imbalance," *Biomed Pharmacother, vol.* 124, p. 109849.

[11.]	L.Y. Dong, S. Li, Y.L. Zhen, Y.N. Wang, X. Shao, and Z.G. Luo. "Cardioprotection of vitexin on myocardial ischemia/reperfusion injury in rat via regulating inflammatory cytokines and MAPK pathway," *Am J Chin Med, vol.* 41, no. 6, pp. 1251-1266.

[12.]	X. Che, X. Wang, J. Zhang, et al. "Vitexin exerts cardioprotective effect on chronic myocardial ischemia/reperfusion injury in rats via inhibiting myocardial apoptosis and lipid peroxidation," *Am J Transl Res, vol.* 8, no. 8, pp. 3319-3328.

[13.]	Y. Yu, N. Xing, X. Xu, et al. "Tournefolic acid B, derived from Clinopodium chinense (Benth.) Kuntze, protects against myocardial ischemia/reperfusion injury by inhibiting endoplasmic reticulum stress-regulated apoptosis via PI3K/AKT pathways," *Phytomedicine, vol.* 52, pp. 178-186.

[14.]	T.J. Zhang, R.X. Guo, X. Li, Y.W. Wang, and Y.J. Li. "Tetrandrine cardioprotection in ischemia-reperfusion (I/R) injury via JAK3/STAT3/Hexokinase II," *Eur J Pharmacol, vol.* 813, pp. 153-160.

[15.]	W. Qian, X. Xiong, Z. Fang, H. Lu, and Z. Wang. "Protective effect of tetramethylpyrazine on myocardial ischemia-reperfusion injury," *Evid Based Complement Alternat Med, vol.* 2014, p. 107501.

[16.]	Z. Tang, C. Yang, B. Zuo, et al. "Taxifolin protects rat against myocardial ischemia/reperfusion injury by modulating the mitochondrial apoptosis pathway," *PeerJ, vol.* 7, p. e6383.

[17.]	Y. Zhang, L. Wei, D. Sun, et al. "Tanshinone IIA pretreatment protects myocardium against ischaemia/reperfusion injury through the phosphatidylinositol 3-kinase/Akt-dependent pathway in diabetic rats," *Diabetes Obes Metab, vol.* 12, no. 4, pp. 316-322.

[18.]	Y. Pan, J.X. Qian, S.Q. Lu, et al. "Protective effects of tanshinone IIA sodium sulfonate on ischemia-reperfusion-induced myocardial injury in rats," *Iran J Basic Med Sci, vol.* 20, no. 3, pp. 308-315.

[19.]	Y. Zhuo, R. Yuan, X. Chen, et al. "Tanshinone I exerts cardiovascular protective effects in vivo and in vitro through inhibiting necroptosis via Akt/Nrf2 signaling pathway," *Chin Med, vol.* 16, no. 1, p. 48.

[20.]	L.J. Xu, R.C. Chen, X.Y. Ma, Y. Zhu, G.B. Sun, and X.B. Sun. "Scutellarin protects against myocardial ischemia-reperfusion injury by suppressing NLRP3 inflammasome activation," *Phytomedicine, vol.* 68, p. 153169.

[21.]	Z. Wang, J. Yu, J. Wu, et al. "Scutellarin protects cardiomyocyte ischemia-reperfusion injury by reducing apoptosis and oxidative stress," *Life Sci, vol.* 157, pp. 200-207.

[22.]	H. Liu, W. Liu, H. Qiu, et al. "Salvianolic acid B protects against myocardial ischaemia-reperfusion injury in rats via inhibiting high mobility group box 1 protein expression through the PI3K/Akt signalling pathway," *Naunyn Schmiedebergs Arch Pharmacol, vol.* 393, no. 8, pp. 1527-1539.

[23.]	H. Fan, L. Yang, F. Fu, et al. "Cardioprotective effects of salvianolic Acid a on myocardial ischemia-reperfusion injury in vivo and in vitro," *Evid Based Complement Alternat Med, vol.* 2012, p. 508938.

[24.]	T. Xu, X. Wu, Q. Chen, et al. "The anti-apoptotic and cardioprotective effects of salvianolic acid a on rat cardiomyocytes following ischemia/reperfusion by DUSP-mediated regulation of the ERK1/2/JNK pathway," *PLoS One, vol.* 9, no. 7, p. e102292.

[25.]	M.Y. Sun, D.S. Ma, S. Zhao, L. Wang, C.Y. Ma, and Y. Bai. "Salidroside mitigates hypoxia/reoxygenation injury by alleviating endoplasmic reticulum stress‑induced apoptosis in H9c2 cardiomyocytes," *Mol Med Rep, vol.* 18, no. 4, pp. 3760-3768.

[26.]	J. Han, D. Wang, L. Ye, et al. "Rosmarinic Acid Protects against Inflammation and Cardiomyocyte Apoptosis during Myocardial Ischemia/Reperfusion Injury by Activating Peroxisome Proliferator-Activated Receptor Gamma," *Front Pharmacol, vol.* 8, p. 456.

[27.]	Z. Xing, Q. He, Y. Xiong, and X. Zeng. "Systematic Pharmacology Reveals the Antioxidative Stress and Anti-Inflammatory Mechanisms of Resveratrol Intervention in Myocardial Ischemia-Reperfusion Injury," *Evid Based Complement Alternat Med, vol.* 2021, p. 5515396.

[28.]	X. Zhang, L.F. Huang, L. Hua, H.K. Feng, and B. Shen. "Resveratrol protects myocardial apoptosis induced by ischemia-reperfusion in rats with acute myocardial infarction via blocking P13K/Akt/e-NOS pathway," *Eur Rev Med Pharmacol Sci, vol.* 23, no. 4, pp. 1789-1796.

[29.]	M. Tian, Y. Xie, Y. Meng, et al. "Resveratrol protects cardiomyocytes against anoxia/reoxygenation via dephosphorylation of VDAC1 by Akt-GSK3 β pathway," *Eur J Pharmacol, vol.* 843, pp. 80-87.

[30.]	H. Xu, J. Cheng, X. Wang, et al. "Resveratrol pretreatment alleviates myocardial ischemia/reperfusion injury by inhibiting STIM1-mediated intracellular calcium accumulation," *J Physiol Biochem, vol.* 75, no. 4, pp. 607-618.

[31.]	L. Yang, Y. Zhang, M. Zhu, et al. "Resveratrol attenuates myocardial ischemia/reperfusion injury through up-regulation of vascular endothelial growth factor B," *Free Radic Biol Med, vol.* 101, pp. 1-9.

[32.]	M. Ding, Y. Wang, D. Sun, et al. "Punicalagin Pretreatment Attenuates Myocardial Ischemia-Reperfusion Injury via Activation of AMPK," *Am J Chin Med, vol.* 45, no. 1, pp. 53-66.

[33.]	H.X. Xu, W. Pan, J.F. Qian, F. Liu, H.Q. Dong, and Q.J. Liu. "MicroRNA‑21 contributes to the puerarin‑induced cardioprotection via suppression of apoptosis and oxidative stress in a cell model of ischemia/reperfusion injury," *Mol Med Rep, vol.* 20, no. 1, pp. 719-727.

[34.]	R. Huang, J. Shu, X. Dai, Y. Liu, F. Yu, and G. Shi. "The protective effect of polyphyllin I on myocardial ischemia/reperfusion injury in rats," *Ann Transl Med, vol.* 8, no. 10, p. 644.

[35.]	K. Ning, L. Jiang, T. Hu, X. Wang, A. Liu, and Y. Bao. "ATP-Sensitive Potassium Channels Mediate the Cardioprotective Effect of Panax notoginseng Saponins against Myocardial Ischaemia-Reperfusion Injury and Inflammatory Reaction," *Biomed Res Int, vol.* 2020, p. 3039184.

[36.]	C.F. Tsai, H.H. Su, K.M. Chen, et al. "Paeonol Protects Against Myocardial Ischemia/Reperfusion-Induced Injury by Mediating Apoptosis and Autophagy Crosstalk," *Front Pharmacol, vol.* 11, p. 586498.

[37.]	F. Wu, B. Ye, X. Wu, et al. "Paeoniflorin on Rat Myocardial Ischemia Reperfusion Injury of Protection and Mechanism Research," *Pharmacology, vol.* 105, no. 5-6, pp. 281-288.

[38.]	L. Liu, Y. Wu, and X. Huang. "Orientin protects myocardial cells against hypoxia-reoxygenation injury through induction of autophagy," *Eur J Pharmacol, vol.* 776, pp. 90-98.

[39.]	C. Lu, C. Chen, A. Chen, et al. "Oridonin Attenuates Myocardial Ischemia/Reperfusion Injury via Downregulating Oxidative Stress and NLRP3 Inflammasome Pathway in Mice," *Evid Based Complement Alternat Med, vol.* 2020, p. 7395187.

[40.]	X. Huang, Y. Wang, Y. Wang, L. Yang, J. Wang, and Y. Gao. "Ophiopogonin D Reduces Myocardial Ischemia-Reperfusion Injury via Upregulating CYP2J3/EETs in Rats," *Cell Physiol Biochem, vol.* 49, no. 4, pp. 1646-1658.

[41.]	B. Ye, X. Chen, S. Dai, et al. "Emodin alleviates myocardial ischemia/reperfusion injury by inhibiting gasdermin D-mediated pyroptosis in cardiomyocytes," *Drug Des Devel Ther, vol.* 13, pp. 975-990.

[42.]	Y. Qiu, N. Cong, M. Liang, Y. Wang, and J. Wang. "Systems Pharmacology Dissection of the Protective Effect of Myricetin Against Acute Ischemia/Reperfusion-Induced Myocardial Injury in Isolated Rat Heart," *Cardiovasc Toxicol, vol.* 17, no. 3, pp. 277-286.

[43.]	F. He, B.L. Xu, C. Chen, et al. "Methylophiopogonanone A suppresses ischemia/reperfusion-induced myocardial apoptosis in mice via activating PI3K/Akt/eNOS signaling pathway," *Acta Pharmacol Sin, vol.* 37, no. 6, pp. 763-771.

[44.]	Y.C. Jin, K.J. Kim, Y.M. Kim, et al. "Anti-apoptotic effect of magnolol in myocardial ischemia and reperfusion injury requires extracellular signal-regulated kinase1/2 pathways in rat in vivo," *Exp Biol Med (Maywood), vol.* 233, no. 10, pp. 1280-1288.

[45.]	T. Xu, D. Li, and D. Jiang. "Targeting cell signaling and apoptotic pathways by luteolin: cardioprotective role in rat cardiomyocytes following ischemia/reperfusion," *Nutrients, vol.* 4, no. 12, pp. 2008-2019.

[46.]	X. Zhang, Q. Du, Y. Yang, et al. "The protective effect of Luteolin on myocardial ischemia/reperfusion (I/R) injury through TLR4/NF-κB/NLRP3 inflammasome pathway," *Biomed Pharmacother, vol.* 91, pp. 1042-1052.

[47.]	M. Zhou, L. Liu, W. Wang, et al. "Role of licochalcone C in cardioprotection against ischemia/reperfusion injury of isolated rat heart via antioxidant, anti-inflammatory, and anti-apoptotic activities," *Life Sci, vol.* 132, pp. 27-33.

[48.]	R. Xiao, A.L. Xiang, H.B. Pang, and K.Q. Liu. "Hyperoside protects against hypoxia/reoxygenation induced injury in cardiomyocytes by suppressing the Bnip3 expression," *Gene, vol.* 629, pp. 86-91.

[49.]	Y.H. Pei, J. Chen, L. Xie, et al. "Hydroxytyrosol Protects against Myocardial Ischemia/Reperfusion Injury through a PI3K/Akt-Dependent Mechanism," *Mediators Inflamm, vol.* 2016, p. 1232103.

[50.]	J. Ye, S. Lu, M. Wang, et al. "Hydroxysafflor Yellow A Protects Against Myocardial Ischemia/Reperfusion Injury via Suppressing NLRP3 Inflammasome and Activating Autophagy," *Front Pharmacol, vol.* 11, p. 1170.

[51.]	J.X. Ye, M. Wang, R.Y. Wang, et al. "Hydroxysafflor yellow A inhibits hypoxia/reoxygenation-induced cardiomyocyte injury via regulating the AMPK/NLRP3 inflammasome pathway," *Int Immunopharmacol, vol.* 82, p. 106316.

[52.]	J. Ye, R. Wang, M. Wang, et al. "Hydroxysafflor Yellow A Ameliorates Myocardial Ischemia/Reperfusion Injury by Suppressing Calcium Overload and Apoptosis," *Oxid Med Cell Longev, vol.* 2021, p. 6643615.

[53.]	M.P. Wu, Y.S. Zhang, Q.M. Zhou, J. Xiong, Y.R. Dong, and C. Yan. "Higenamine protects ischemia/reperfusion induced cardiac injury and myocyte apoptosis through activation of β2-AR/PI3K/AKT signaling pathway," *Pharmacol Res, vol.* 104, pp. 115-123.

[54.]	L. Chang, R. Shi, X. Wang, and Y. Bao. "Gypenoside A protects ischemia/reperfusion injuries by suppressing miR-143-3p level via the activation of AMPK/Foxo1 pathway," *Biofactors, vol.* 46, no. 3, pp. 432-440.

[55.]	C. Ren, Y.R. Bao, X.S. Meng, Y.P. Diao, and T.G. Kang. "Comparison of the protective effects of ferulic acid and its drug-containing plasma on primary cultured neonatal rat cardiomyocytes with hypoxia/reoxygenation injury," *Pharmacogn Mag, vol.* 9, no. 35, pp. 202-209.

[56.]	T. Lai, Y. Shen, C. Chen, et al. "Glycyrrhizic acid ameliorates myocardial ischemia-reperfusion injury in rats through inhibiting endoplasmic reticulum stress," *Eur J Pharmacol, vol.* 908, p. 174353.

[57.]	X. Chen. "Cardiovascular protection by ginsenosides and their nitric oxide releasing action," *Clin Exp Pharmacol Physiol, vol.* 23, no. 8, pp. 728-732.

[58.]	L. Li, Y. Wang, R. Guo, et al. "Ginsenoside Rg3-loaded, reactive oxygen species-responsive polymeric nanoparticles for alleviating myocardial ischemia-reperfusion injury," *J Control Release, vol.* 317, pp. 259-272.

[59.]	Y. Shi, B. Han, X. Yu, S. Qu, and D. Sui. "Ginsenoside Rb3 ameliorates myocardial ischemia-reperfusion injury in rats," *Pharm Biol, vol.* 49, no. 9, pp. 900-906.

[60.]	L. Li, C.S. Pan, L. Yan, et al. "Ginsenoside Rg1 Ameliorates Rat Myocardial Ischemia-Reperfusion Injury by Modulating Energy Metabolism Pathways," *Front Physiol, vol.* 9, p. 78.

[61.]	Y.C. Cui, C.S. Pan, L. Yan, et al. "Ginsenoside Rb1 protects against ischemia/reperfusion-induced myocardial injury via energy metabolism regulation mediated by RhoA signaling pathway," *Sci Rep, vol.* 7, p. 44579.

[62.]	Q. Xiang, M. Wu, L. Zhang, et al. "Gerontoxanthone I and Macluraxanthone Induce Mitophagy and Attenuate Ischemia/Reperfusion Injury," *Front Pharmacol, vol.* 11, p. 452.

[63.]	W.L. Jiang, F.H. Fu, B.M. Xu, J.W. Tian, H.B. Zhu, and H. Jian. "Cardioprotection with forsythoside B in rat myocardial ischemia-reperfusion injury: relation to inflammation response," *Phytomedicine, vol.* 17, no. 8-9, pp. 635-639.

[64.]	Z. Huang, Y. Liu, and X. Huang. "Formononetin may protect aged hearts from ischemia/reperfusion damage by enhancing autophagic degradation," *Mol Med Rep, vol.* 18, no. 6, pp. 4821-4830.

[65.]	Y. Hao, Y. Sun, C. Xu, et al. "Improvement of contractile function in isolated cardiomyocytes from ischemia-reperfusion rats by ginkgolide B pretreatment," *J Cardiovasc Pharmacol, vol.* 54, no. 1, pp. 3-9.

[66.]	W.Q. Rang, Y.H. Du, C.P. Hu, et al. "Protective effects of evodiamine on myocardial ischemia-reperfusion injury in rats," *Planta Med, vol.* 70, no. 12, pp. 1140-1143.

[67.]	D. Li, N. Lu, J. Han, et al. "Eriodictyol Attenuates Myocardial Ischemia-Reperfusion Injury through the Activation of JAK2," *Front Pharmacol, vol.* 9, p. 33.

[68.]	J. Nan, C. Nan, J. Ye, et al. "EGCG protects cardiomyocytes against hypoxia-reperfusion injury through inhibition of OMA1 activation," *J Cell Sci, vol.* 132, no. 2.

[69.]	H. Chang, X.Y. Chu, J. Zou, and T.H. Chang. "Effect of dl-praeruptorin A on desmin and vimentin content in rat ischemia/reperfusion myocardiocytes," *Chin Med J (Engl), vol.* 120, no. 24, pp. 2256-2259.

[70.]	C. Yi, L. Si, J. Xu, J. Yang, Q. Wang, and X. Wang. "Effect and mechanism of asiatic acid on autophagy in myocardial ischemia-reperfusion injury in vivo and in vitro," *Exp Ther Med, vol.* 20, no. 5, p. 54.

[71.]	L. Wei, X. Sun, X. Qi, Y. Zhang, Y. Li, and Y. Xu. "Dihydromyricetin Ameliorates Cardiac Ischemia/Reperfusion Injury through Sirt3 Activation," *Biomed Res Int, vol.* 2019, p. 6803943.

[72.]	G. Fan, J. Yu, P.F. Asare, et al. "Danshensu alleviates cardiac ischaemia/reperfusion injury by inhibiting autophagy and apoptosis via activation of mTOR signalling," *J Cell Mol Med, vol.* 20, no. 10, pp. 1908-1919.

[73.]	J. Yu, L. Wang, M. Akinyi, et al. "Danshensu protects isolated heart against ischemia reperfusion injury through activation of Akt/ERK1/2/Nrf2 signaling," *Int J Clin Exp Med, vol.* 8, no. 9, pp. 14793-14804.

[74.]	Y. Wang, Q. Wang, L. Zhang, et al. "Coptisine protects cardiomyocyte against hypoxia/reoxygenation-induced damage via inhibition of autophagy," *Biochem Biophys Res Commun, vol.* 490, no. 2, pp. 231-238.

[75.]	S. Sun and P. Wang. "Coptisine alleviates ischemia/reperfusion-induced myocardial damage by regulating apoptosis-related proteins," *Tissue Cell, vol.* 66, p. 101392.

[76.]	M. Jiang, J. Ni, Y. Cao, X. Xing, Q. Wu, and G. Fan. "Astragaloside IV Attenuates Myocardial Ischemia-Reperfusion Injury from Oxidative Stress by Regulating Succinate, Lysophospholipid Metabolism, and ROS Scavenging System," *Oxid Med Cell Longev, vol.* 2019, p. 9137654.

[77.]	J. Si, N. Wang, H. Wang, et al. "HIF-1α signaling activation by post-ischemia treatment with astragaloside IV attenuates myocardial ischemia-reperfusion injury," *PLoS One, vol.* 9, no. 9, p. e107832.

[78.]	L. Tu, C.S. Pan, X.H. Wei, et al. "Astragaloside IV protects heart from ischemia and reperfusion injury via energy regulation mechanisms," *Microcirculation, vol.* 20, no. 8, pp. 736-747.

[79.]	H. Huang, S. Lai, Q. Wan, W. Qi, and J. Liu. "Astragaloside IV protects cardiomyocytes from anoxia/reoxygenation injury by upregulating the expression of Hes1 protein," *Can J Physiol Pharmacol, vol.* 94, no. 5, pp. 542-553.

[80.]	Y. Wu, Z. Fan, Z. Chen, et al. "Astragaloside IV protects human cardiomyocytes from hypoxia/reoxygenation injury by regulating miR-101a," *Mol Cell Biochem, vol.* 470, no. 1-2, pp. 41-51.

[81.]	J.J. Yang, X.H. Zhang, X.H. Ma, et al. "Astragaloside IV enhances GATA-4 mediated myocardial protection effect in hypoxia/reoxygenation injured H9c2 cells," *Nutr Metab Cardiovasc Dis, vol.* 30, no. 5, pp. 829-842.

[82.]	X. Tang, J. Liu, W. Dong, et al. "The cardioprotective effects of citric Acid and L-malic Acid on myocardial ischemia/reperfusion injury," *Evid Based Complement Alternat Med, vol.* 2013, p. 820695.

[83.]	M. Wang, R.Y. Wang, J.H. Zhou, X.H. Xie, G.B. Sun, and X.B. Sun. "Calenduloside E Ameliorates Myocardial Ischemia-Reperfusion Injury through Regulation of AMPK and Mitochondrial OPA1," *Oxid Med Cell Longev, vol.* 2020, p. 2415269.

[84.]	J.H. Jia, K.P. Chen, S.X. Chen, K.Z. Liu, T.L. Fan, and Y.C. Chen. "Breviscapine, a traditional Chinese medicine, alleviates myocardial ischaemia reperfusion injury in diabetic rats," *Acta Cardiol, vol.* 63, no. 6, pp. 757-762.

[85.]	Y. Bai, Z. Li, W. Liu, D. Gao, M. Liu, and P. Zhang. "Biochanin A attenuates myocardial ischemia/reperfusion injury through the TLR4/NF-κB/NLRP3 signaling pathway," *Acta Cir Bras, vol.* 34, no. 11, p. e201901104.

[86.]	B. Xiao, X. Huang, Q. Wang, and Y. Wu. "Beta-Asarone Alleviates Myocardial Ischemia-Reperfusion Injury by Inhibiting Inflammatory Response and NLRP3 Inflammasome Mediated Pyroptosis," *Biol Pharm Bull, vol.* 43, no. 7, pp. 1046-1051.

[87.]	Z. Qin-Wei and L.I. Yong-Guang. "Berberine attenuates myocardial ischemia reperfusion injury by suppressing the activation of PI3K/AKT signaling," *Exp Ther Med, vol.* 11, no. 3, pp. 978-984.

[88.]	P. Zhang, X. Liu, G. Huang, C. Bai, Z. Zhang, and H. Li. "Barbaloin pretreatment attenuates myocardial ischemia-reperfusion injury via activation of AMPK," *Biochem Biophys Res Commun, vol.* 490, no. 4, pp. 1215-1220.

[89.]	L. Qiu, J. Chen, J. Lin, D. Wo, J. Chu, and J. Peng. "Baicalin alleviates H2O2‑induced injury of H9c2 cardiomyocytes through suppression of the Wnt/β‑catenin signaling pathway," *Mol Med Rep, vol.* 16, no. 6, pp. 9251-9255.

[90.]	L. Song, H. Yang, H.X. Wang, et al. "Inhibition of 12/15 lipoxygenase by baicalein reduces myocardial ischemia/reperfusion injury via modulation of multiple signaling pathways," *Apoptosis, vol.* 19, no. 4, pp. 567-580.

[91.]	S. Yang, G. Chou, and Q. Li. "Cardioprotective role of azafrin in against myocardial injury in rats via activation of the Nrf2-ARE pathway," *Phytomedicine, vol.* 47, pp. 12-22.

[92.]	D. Qu, J. Han, H. Ren, et al. "Cardioprotective Effects of Astragalin against Myocardial Ischemia/Reperfusion Injury in Isolated Rat Heart," *Oxid Med Cell Longev, vol.* 2016, p. 8194690.

[93.]	M. Wang, Y. Tian, Y.Y. Du, et al. "Protective effects of Araloside C against myocardial ischaemia/reperfusion injury: potential involvement of heat shock protein 90," *J Cell Mol Med, vol.* 21, no. 9, pp. 1870-1880.

[94.]	P. Wang, J. Sun, S. Lv, T. Xie, and X. Wang. "Apigenin Alleviates Myocardial Reperfusion Injury in Rats by Downregulating miR-15b," *Med Sci Monit, vol.* 25, pp. 2764-2776.

[95.]	Z. Zhou, Y. Zhang, L. Lin, and J. Zhou. "Apigenin suppresses the apoptosis of H9C2 rat cardiomyocytes subjected to myocardial ischemia‑reperfusion injury via upregulation of the PI3K/Akt pathway," *Mol Med Rep, vol.* 18, no. 2, pp. 1560-1570.

[96.]	W. Li, L. Chen, and Y. Xiao. "Apigenin protects against ischemia-/hypoxia-induced myocardial injury by mediating pyroptosis and apoptosis," *In Vitro Cell Dev Biol Anim, vol.* 56, no. 4, pp. 307-312.

[97.]	X.N. Xu, Y. Jiang, L.Y. Yan, et al. "Aesculin suppresses the NLRP3 inflammasome-mediated pyroptosis via the Akt/GSK3β/NF-κB pathway to mitigate myocardial ischemia/reperfusion injury," *Phytomedicine, vol.* 92, p. 153687.

[98.]	X. Lv, T. Xu, Q. Wu, et al. "6-Gingerol Activates PI3K/Akt and Inhibits Apoptosis to Attenuate Myocardial Ischemia/Reperfusion Injury," *Evid Based Complement Alternat Med, vol.* 2018, p. 9024034.

[99.]	T. Xu, G. Qin, W. Jiang, Y. Zhao, Y. Xu, and X. Lv. "6-Gingerol Protects Heart by Suppressing Myocardial Ischemia/Reperfusion Induced Inflammation via the PI3K/Akt-Dependent Mechanism in Rats," *Evid Based Complement Alternat Med, vol.* 2018, p. 6209679.

[100.]	W. Zhang, X. Liu, Y. Jiang, N. Wang, F. Li, and H. Xin. "6-Gingerol Attenuates Ischemia-Reperfusion-Induced Cell Apoptosis in Human AC16 Cardiomyocytes through HMGB2-JNK1/2-NF-κB Pathway," *Evid Based Complement Alternat Med, vol.* 2019, p. 8798653.

[101.]	J.S. Hwa, Y.C. Jin, Y.S. Lee, et al. "2-methoxycinnamaldehyde from Cinnamomum cassia reduces rat myocardial ischemia and reperfusion injury in vivo due to HO-1 induction," *J Ethnopharmacol, vol.* 139, no. 2, pp. 605-615.
